# Evaluating the safety, tolerability, pharmacokinetics and efficacy of clofazimine in cryptosporidiosis (CRYPTOFAZ): study protocol for a randomized controlled trial

**DOI:** 10.1186/s13063-018-2846-6

**Published:** 2018-08-23

**Authors:** Patrick Nachipo, David Hermann, Gerald Quinnan, Melita A. Gordon, Wesley C. Van Voorhis, Pui-Ying Iroh Tam

**Affiliations:** 1 University of Malawi College of Medicine, Blantyre, Malawi; 2 Bill and Melinda Gates Foundation, Seattle, WA USA; 3 Emmes Corporation, Rockville, MD USA; 4 University of Liverpool, Liverpool, UK; 5 Malawi-Liverpool Wellcome Trust Clinical Research Programme, Blantyre, Malawi; 6 University of Washington, Seattle, WA USA; 7 Liverpool School of Tropical Medicine, Liverpool, UK

**Keywords:** Cryptosporidium, Diarrhea, HIV, Clofazimine, Lamprene, Immunosuppression

## Abstract

**Background:**

Cryptosporidium infection and diarrhea (cryptosporidiosis) is a life-threatening infection in persons with HIV and also in children of 6–18 months of age in the developing world. To date, only nitazoxanide is licensed for treatment of cryptosporidiosis, and only in persons after the first year of life and with healthy immune systems. Clofazimine (CFZ: Lamprene®), an established drug that has been used for leprosy for more than 50 years, recently has been described as effective against Cryptosporidium in vitro and in mouse infections. The efficacy and pharmacokinetics of CFZ in vivo*,* in HIV-infected patients with cryptosporidial diarrhea are not known.

**Methods:**

CRYPTOFAZ includes a randomized, double-blind, placebo-controlled study of the safety, tolerability and Cryptosporidium inhibitory activity of orally administered CFZ in subjects with HIV infection and chronic diarrhea with Cryptosporidium. An additional open label aspect of the study will compare the pharmacokinetics (PK) of orally administered CFZ in HIV-infected individuals with and without Cryptosporidium-associated diarrhea. The study will recruit a total of 66 subjects. Study participants will be given either CFZ or a placebo for 5 days while in hospital and will be followed up after discharge. Cryptosporidium will be diagnosed by quantitative PCR as the definitive test and by stool ELISA, which will also be used to quantify the shedding of Cryptosporidium in stool. PK will be studied on plasma and stool samples. Primary endpoints include reduction in the number of Cryptosporidium shed in stools over a 5-day period and compared to placebo recipients and the PK of CFZ in plasma assessed by area under the curve, peak plasma concentration, and half-life (T ½) determined after the last dose.

**Discussion:**

This study provides an opportunity to explore a possible treatment option for HIV-infected patients with cryptosporidial diarrhea, who, as of now in Malawi and most of sub-Saharan Africa, do not have a definitive treatment apart from supportive care. The strength of this study lies in it being a randomized, double-blind, placebo-controlled trial. If shown to be effective and safe, the findings will also lay a foundation for a future study of the use of CFZ in children 6–18 months of age.

**Trial registration:**

ClinicalTrials.gov, NCT03341767. Registered on 14 November 2017.

**Electronic supplementary material:**

The online version of this article (10.1186/s13063-018-2846-6) contains supplementary material, which is available to authorized users.

## Background

Cryptosporidium infection and diarrhea (cryptosporidiosis) is a life-threatening infection in persons with HIV and also in children 6–18 months of age in the developing world [1, 2]. Two main species of Cryptosporidium cause infections in humans; *Cryptosporidium hominis*, causing the majority of the infections, and *Cryptosporidium parvum* causing 20–30% of the infections [2]. To date, only nitazoxanide is licensed for treatment of cryptosporidiosis, and only in persons after the first year of life and with healthy immune systems. A randomized trial in malnourished children that were hospitalized with cryptosporidiosis and diarrhea showed only 30% efficacy of nitazoxanide versus placebo in the treatment of cryptosporidiosis in HIV seronegative, but not seropositive children [3]. It is not licensed for use in HIV-infected and immunocompromised patients. One study claimed benefits of using nitazoxanide in HIV-infected adult patients with CD4+ cell counts greater than 50 cells/μL by administering high doses of the drug [4]. In studies examining mortality as the endpoint, nitazoxanide has not shown any benefits in HIV-infected and immunocompromised patients with cryptosporidiosis compared to placebo [3, 5, 6].

Recently, an old drug, clofazimine (CFZ, sold under the brand name Lamprene®) has been described as effective against Cryptosporidium in vitro, and has been shown to eliminate *C. parvum* in a mouse model [7]. Clofazimine has been in use for more than 50 years for treatment of leprosy, and remains on the World Health Organization (WHO) essential drug list. Clofazimine is also used as part of a WHO regimen to treat multi-drug resistant *Mycobacterium tuberculosis*. The pharmacokinetics (PK) of CFZ administration have been reported after a single dose and multiple dosing, but never in individuals with diarrhea or HIV infection.

Discovery of an effective drug for cryptosporidiosis that is safe to use in HIV-infected patients with advanced immunosuppression and in children under 1 year of age will benefit these groups of patients, who as of now do not have any effective treatment options. This clinical trial will evaluate the safety, tolerability, PK and efficacy of CFZ in HIV-infected adults with cryptosporidiosis.

## Methods/design

### Study hypotheses

This study (Additional file 1) is divided into two parts. The first part, part A, is a phase 2A study that assesses safety, tolerability and anti-cryptosporidial activity of orally administered CFZ. For part A, we hypothesize that CFZ (Lamprene®) orally administered daily for 5 consecutive days, will be safe and well-tolerated and will significantly reduce Cryptosporidium concentration in the first collected stool of the day over a 5-day period, as measured by quantitative PCR (qPCR) 18S rRNA specific to Cryptosporidium species. This will be tested by analysis of covariance (ANCOVA) in comparison to comparable placebo-treated HIV-infected controls with chronic diarrhea and Cryptosporidium infection treated according to protocol (ATP).

The second part, part B, is a phase 2A study of the PK of orally administered CFZ (Lamprene®) in HIV-infected adults without diarrhea or Cryptosporidium infection. The PK of CFZ in blood and stool in this population will be compared to those in the HIV-infected adults with diarrhea and Cryptosporidium infection. For this part, we hypothesize that the PK of CFZ will be similar in HIV-infected adults with and without diarrhea and Cryptosporidium infection.

### Study objectives

The primary objectives for part A are to evaluate whether there is a reduction in Cryptosporidium fecal shedding following CFZ administration relative to placebo in HIV-infected adults with diarrhea and Cryptosporidium infection; and to investigate the safety and tolerability of CFZ when orally administered daily for 5 consecutive days in this population. For part B, the objective is to investigate the PK of CFZ in HIV-infected adults with Cryptosporidium infection and diarrhea compared to the PK in HIV-infected subjects without Cryptosporidium infection or diarrhea.

### Study design

This is a two-part study. Part A is a randomized, double-blind, placebo-controlled study of the safety, tolerability and Cryptosporidium inhibitory activity of orally administered CFZ in subjects with HIV infection and chronic diarrhea (for at least 3 days’ duration), with Cryptosporidium infection demonstrated by stool testing. Part B is an open label study, intended to compare the PK of orally administered CFZ in HIV-infected individuals with and without Cryptosporidium-associated diarrhea.

### End points

#### Primary endpoints

The primary endpoints are:

1. Safety assessments collected throughout the follow up period: adverse events (AEs), serious adverse events (SAEs), vital signs, 12-lead electrocardiogram (ECG), physical examinations (PE), and clinical laboratory evaluations.
2. The reduction in the (log) number of Cryptosporidium shed in stools in the first collected stool of the day over a 5-day period and compared to placebo recipients, as measured by qPCR in stool samples and analyzed by mixed-effect analysis of covariance (ANCOVA) in subjects treated ATP (part A only).
3. Pharmacokinetics of CFZ in plasma: area under the curve (AUC), peak plasma concentration (Cmax), and time to reach Cmax (Tmax) on the second and last dose days; half-life (T ½) determined after the last dose.
4. The amount of CFZ in stool on day 2 (2nd dose day), day 5 (last dose day), and day 6 (concentration of CFZ in stool before discharge).

#### Secondary endpoints

The secondary endpoints are:

1. The reduction in the (log) number of Cryptosporidium shed in stools in the first collected stool of the day over a 5-day period and compared to placebo recipients in the intention to treat (ITT) subject population
2. The reduction in total daily Cryptosporidium shedding over a 6-day period in subjects treated ATP (part A only)
3. The reduction in total daily Cryptosporidium shedding over a 6-day period in comparison to placebo controls in the ITT population
4. The reduction in severity of diarrhea over a 6-day period in comparison to placebo controls in subjects treated ATP

### Study site

The study take place at Queen Elizabeth Central Hospital (QECH) in Blantyre, Malawi. It is the main referral hospital for the southern region in Malawi. There is an outpatient Gateway Clinic close by the hospital, that screens and either manages or refers outpatients, who may be self-referred or referred from primary Health Centers, before they are seen at the tertiary hospital; some additional subjects will be recruited from the Gateway Clinic.

### Sample size

The sample size for part A was planned based on the goal that demonstration of a significant inhibition of Cryptosporidium shedding should be possible in a relatively small number of subjects, if the drug is to be worth further development efforts. Based on results in a calf model with a controlled challenge, the sample size required would be very small (e.g. 10 per group) if the variability of shedding in the study population planned for enrollment in this study is small. Since the variability in shedding in the planned study population cannot be estimated, a study size of 25 per group (increased to 28 per group to cover dropouts) was planned in case variability is more substantial than expected. An interim analysis is planned after 20 subjects have been randomized and successfully completed the study drug administration phase of the study. An analysis of the primary efficacy endpoint is planned at that time for success. If success is determined, the study will be terminated. If success is not determined, an analysis for futility of continuation will be performed. If the study appears to be adequately powered to achieve significance of the primary efficacy endpoint based on that analysis, it will be continued until full enrollment and follow up.

The second part of this study is empirical, and will include approximately the first 10 CFZ recipients in part A and a new set of 10 recipients without diarrhea or Cryptosporidium infection who will be recruited specifically for part B. Pharmacology of CFZ will be compared in the individuals receiving CFZ in part A with those individuals receiving CFZ in part B. The objective is to develop a comparative description of the absorption and excretion of the drug in the HIV-positive populations with and without Cryptosporidium-associated diarrhea.

### Inclusion/exclusion criteria

#### Part A

##### Inclusion criteria

The subjects to be included will be male or female, aged 18–65 years old, HIV-positive, Cryptosporidium positive by qPCR, and on stable antiretroviral (ARV) treatment for at least 2 weeks. They should weigh more than 35.4 kg and should have chronic diarrhea (defined as a condition of three or more loose stools per day that has persisted for 3 days or longer). If female, they should not be of reproductive potential (post-menopause, or status-post surgical sterilization) or be using, or be willing to use, highly effective contraception (< 1% failure, e.g., intrauterine contraceptive device or injectable contraception). They should be willing and able to provide signed written informed consent (IC) or witnessed documented oral consent in the case of illiteracy, prior to undertaking any trial-related procedures.

##### Exclusion criteria

The exclusion criteria are:

- Any condition for which participation in the study, as judged by the investigator, could compromise the wellbeing of the subject or prevent, limit or confound protocol-specified assessments.
- Fever > 38.0 °C on presentation.
- Evidence of active tuberculosis based on acid fast bacilli staining or GeneXpert testing of sputum or sputum production, fever, and chest x-ray consistent with tuberculosis.
- Critical illness, or in the judgment of the investigator a prognosis that could lead to imminent mortality within 60 days, compromise participation in the trial, or endanger the subject by entering the trial
- History of allergy or hypersensitivity to CFZ
- Significant cardiac arrhythmia requiring medication
- ECG exclusions based on the means from triplicate ECGs performed on day 1:
- Marked prolongation of the QT/QTc interval, e.g., confirmed demonstration of QTcF or QTcB interval > 450 ms
- Pathological Q waves (defined as > 40 ms or depth > 0.4 mV);
- ECG evidence of ventricular pre-excitation
- ECG evidence of complete or incomplete left bundle branch block or right bundle branch block
- ECG evidence of second-degree or third-degree heart block
- Intraventricular conduction delay with QRS duration > 120 ms
- Bradycardia as defined by sinus rate < 50 bpm
- History of additional risk factors for Torsade de Pointes, e.g., heart failure; bradycardia with heart rate (HR) < 50 bpm, untreated hypothyroidism, hypokalemia < 3.2 mEq/L
- Family history of long QT syndrome
- Use of concomitant medications that markedly prolong the QT/QTc interval or are predicted to have drug-drug interactions with CFZ that may lead to toxicity from the partner drug, including amiodarone, amprenavir, atazanavir, bedaquiline, bepridil, chloroquine, chlorpromazine, cisapride, clarithromycin, cyclobenzaprine, darunavir, delamanid, disopyramide dofetilide, domperidone, droperidol, erythromycin, fosamprenavir, halofantrine, haloperidol, ibutilide, indinavir, levomethadyl, lopinavir, mesoridazine, methadone, nelfinavir, pentamidine, pimozide, procainamide, quinidine, ritonavir, simiprinivir, sotalol, sparfloxacin, thioridazine, or tiprinivir
- Pregnant and lactating women
- Use of systemic corticosteroids or anti-cryptosporidial treatments within the preceding 28 days
- Clinically significant laboratory value abnormalities at screening including but not limited to (note: exclusionary results may not be returned until after enrollment but should be confirmed by the time of the beginning of administration of study drug):
- Hemoglobin < 5 g/dL
- Serum potassium < 3.0 mEq/L
- Aspartate aminotransferase (AST) or alanine aminotransferase (ALT) ≥ 3.0 times the upper limit of normal (ULN)

#### Part B

Patients presenting to the HIV or other outpatient clinics at QECH who do not have diarrhea will be offered the opportunity to participate. Subjects will be selected on the basis of matching (demographic) criteria compared to the first 10 subjects weighing > 50 kg who are enrolled and administered the investigational product (IP) (with either CFZ or placebo) in part A, i.e., based on similar age (± 5 years), gender, and stage of HIV infection according to the WHO criteria. Subjects enrolled in part B will need to weigh > 50 kg. Otherwise, except for the absence of diarrhea and Cryptosporidium infection, the eligibility and exclusion criteria will be the same as for subjects enrolled in part A.

### Study phases

There will be three phases in the study: screening (prescreening and screening) and inpatient and outpatient phases.

### Screening phases

**Fig. 1:**
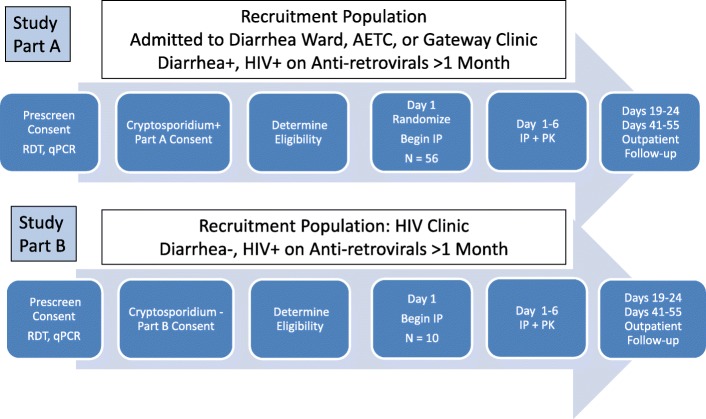
Schematic diagram for study enrollment and conduct. RDT, *Cryptosporidium* rapid diagnostic test; IP, investigational product; PK, pharmacokinetics

The screening phase of the study will include the prescreening and screening phases (Fig. 1). In the prescreening phase, stool is tested for the presence of Cryptosporidium. Consent to participate in the screening and study phases will be obtained after prescreening. After documented informed consent (IC) and enrollment in part A or part B, subjects will be screened for additional eligibility and exclusion criteria. Those subjects who are eligible for the treatment phases of part A or part B will continue in the study. The prescreening and screening phases for parts A and B are expected to last from 1 to 5 days.

### Study phase: inpatient

Subjects participating in parts A and B will be monitored daily during the inpatient phase of the study via examination by a study clinician, solicitation of daily progress reports, monitoring of gastrointestinal (GI), dermatological, and systemic signs and symptoms, reviewing medical conditions, and noting adverse events (AE). Vital signs will be recorded three times daily, and more often, based on the clinician’s judgment. If a subject develops GI symptoms or other expected AEs that cause them to desire discontinuation of treatment, IP administration will be discontinued and the reasons for discontinuation recorded. Blood and stool samples will be collected according to protocol. All stools will be collected in disposable plastic containers for weighing and grading of the stool consistency. The volume will be calculated from the weight, assuming 1 g  = 1 mL. For part A, stools will be graded for presence and severity of diarrhea.

### Study phase: outpatient (parts A and B)

Subjects will be asked to return to the outpatient clinic for one outpatient follow up visit during the interval days 19–24, and one outpatient follow up visit during the interval days 41–55. They will be questioned about the occurrence of any symptoms reflecting expected AEs associated with CFZ treatment and any other intervening illnesses, and blood and stool samples will be collected as per protocol.

### Study treatments, dose, and mode of administration

#### Part A

This part is to assess the safety and tolerability of CFZ when administered to subjects with HIV infection and diarrhea with positive stool test for Cryptosporidium, and the potential inhibitory effect of CFZ treatment on shedding of Cryptosporidium in stool. There will be an initial screening phase and a study phase. For the initial screening phase, subjects presenting with chronic diarrhea and a history of HIV infection with ARV treatment for at least a month will be asked to volunteer to be tested for the presence of Cryptosporidium infection as a screening test for potential participation in the treatment phase. After providing documented IC, subjects will be screened. Subjects testing positive for Cryptosporidium infection will be asked to volunteer for the treatment phase of the study. After consenting to participate for the treatment phase, full eligibility for participation in this phase will be determined.

The complete screening process including prescreening and screening for part A will take 1–5 days. Fully eligible subjects will be admitted to the clinical research ward. Subjects who do not meet all eligibility criteria will be informed of their results and referred for any treatment required, as appropriate. The day of admission to the clinical research ward will be day 1. Subjects will be randomized to the IP and administration of the IP will begin on day 1. Assessments will continue daily during the inpatient phase. The inpatient phase will include IP administration on days 1–5.

On day 6, assessments will be performed and the subjects will be discharged for outpatient follow-up. Subjects will be asked to return to the outpatient clinic for one outpatient follow up visit during the interval days 19–24, and one outpatient follow up visit during the interval days 41–55 unless additional follow up is needed until resolution of ongoing AEs or completion of unexpected pregnancy.

The active IP will be administered orally as 50-mg gelatin capsules of micronized CFZ suspended in an oil-wax base. Two capsules of either CFZ (100 mg) or placebo will be given to subjects weighing 50 kg or more and one capsule for those weighing less. Both the active drug and the placebo are made and provided for this study by Novartis. Study drug will be taken with food and to achieve this uniformly, study subjects will be fed with Plumpy’Soy or Plumpy’Nut nutritional supplement 30 min before each dose, and each dose will be given at least 1.5 h before the next anticipated meal. The doses will be administered at 5:00 a.m., 11:00 a.m., and 5:00 p.m. Accordingly, a total daily dose of 150 mg (body weight < 50 kg) or 300 mg (body weight > 50 kg) will be administered for 5 days. The IP dose was split to minimize the probability of fecal excretion of intact Lamprene gelatin capsules. If clinical research staff members discover that a dose was not administered on time, it will be administered immediately and the actual time noted, and the subsequent dose should not be given any sooner than 4 h after the “late” dose. A “missed/late” dose within an hour of the next dose, will be forgone and the next scheduled dose administered as planned. If a subject misses administration of the IP for ≥ 48 h, treatment will be discontinued.

If a subject leaves the study ward before completion of the scheduled study procedures on day 6, an effort will be made to contact the subject at his/her home to complete CFZ dosing and specimen collection. Subjects who fail to complete the study will be included in the ITT analyses, and any PK data obtained will be included in the PK analyses. If a subject does not return for follow up, efforts will be made to locate the subject to complete the study as an outpatient.

#### Part B

This part is to compare the PK of orally administered CFZ in HIV-infected individuals with and without Cryptosporidium-associated diarrhea and will include individuals in part A who received CFZ and 10 subjects without diarrhea. The 10 subjects in part B will be recruited from the HIV and other outpatient clinics and will not be randomized. They will provide IC and be screened for the presence of Cryptosporidium. If negative, they will provide documented IC, and will be admitted to the Clinical Inpatient Unit. They will receive the active IP, with a dosing schedule similar to part A participants. Since subjects will be admitted specifically for the purpose of this study, completely missed doses are not anticipated. Should a subject discontinue participation before the 4th day of CFZ administration, an additional subject will be recruited. All of the pharmacodynamic data obtained for both the dropout and replacement subjects will be included in data analyses.

## Screening, randomization, and masking procedures

### Screening

#### Part A

Patients who meet the inclusion criteria will be asked to consent to participate in a study that involves initial screening for the presence of Cryptosporidium. After a subject provides consent, a stool sample will be collected and subjected to Cryptosporidium rapid diagnostic test (RDT) and qPCR. It is anticipated that the results of the RDT and qPCR will be available within 8 h and 36 h, respectively. Subjects who are positive by RDT will be offered the opportunity to participate in part A. Subjects who were RDT positive but qPCR negative will be informed of the results and that they will not be eligible to continue in part A. Subjects who were RDT negative but are found to be qPCR positive will be offered the opportunity to participate in part A.

#### Part B

Screening for participation in part B will follow the same process as for participation in part A, except that subjects will be offered the opportunity to participate only if they do not have diarrhea, and stool screening tests (RDT and qPCR) are negative for infection with Cryptosporidium.

### Enrollment and randomization

Subject randomization will occur at the time of initiation of treatment. An unblinded pharmacist will be provided with the treatment assignment codes for preparation of the CFZ or placebo to be given to each subject. A designated individual at the site, i.e. the research pharmacist, will be provided with a code list for emergency unblinding purposes, which will be kept in a secure place.

### Laboratory evaluations

#### Clinical laboratory evaluation

Stool samples will be collected from the subjects enrolled in the study and the following tests will be conducted:

- Cryptosporidium ELISA test (TechLabs Inc.)
- Cryptosporidium DNA by qPCR analysis
- Baseline stool sample DNA will be preserved for later GI TaqMan Array analysis
- Cryptosporidium genotyping for species and subtype
- Cryptosporidium oocysts from day 1 will be evaluated for sensitivity of the individuals’ Cryptosporidium isolate to CFZ in vitro

**Fig. 2:**
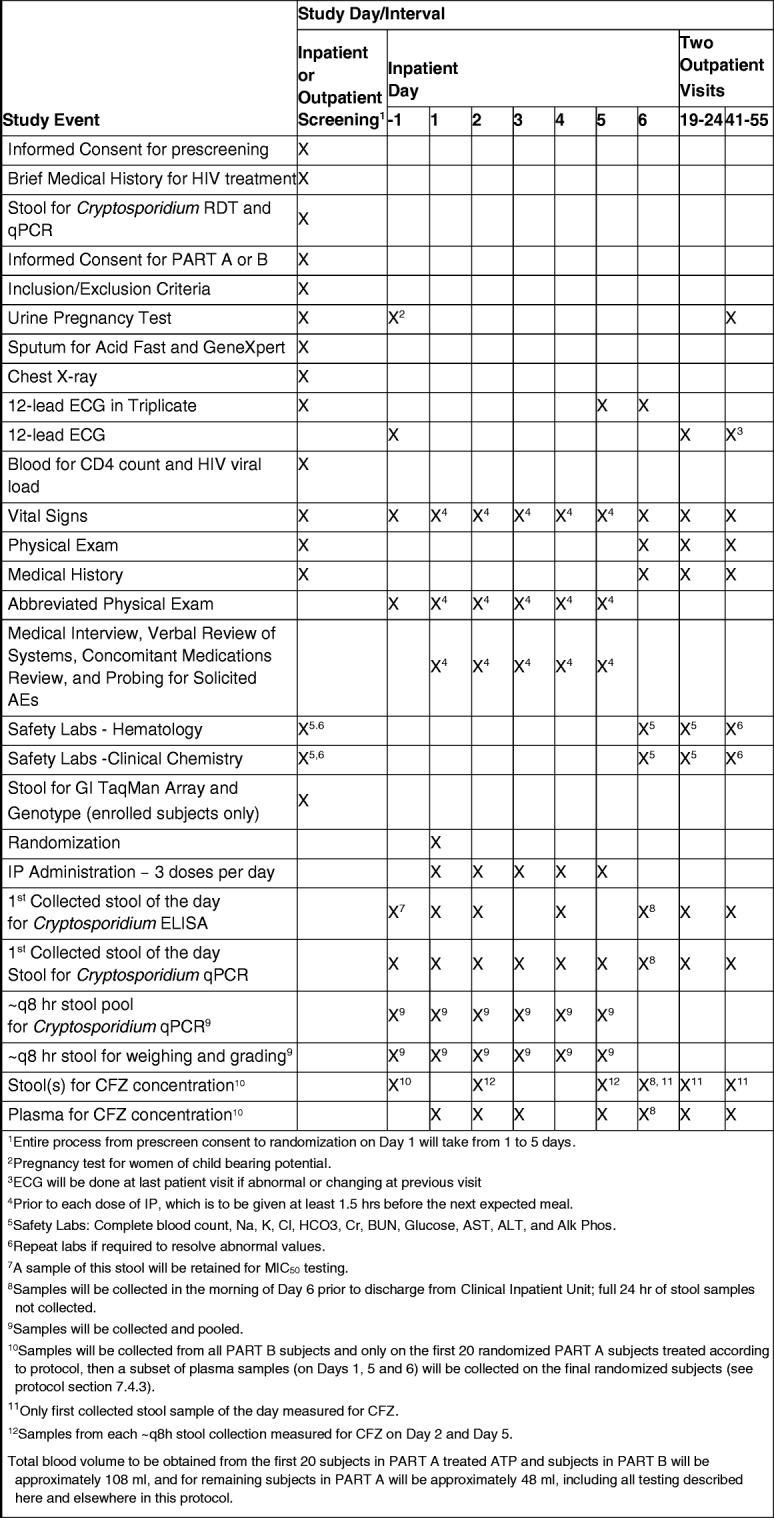
Summary of study events schedule. RDT, *Cryptosporidium* rapid diagnostic test; ECG, electrocardiogram; AEs, adverse events; GI, gastrointestinal; IP, investigational product; ELISA, enzyme-linked immunosorbent assay; CFZ, clofazimine; BUN, blood urea nitrogen; AST, aspartate aminotransferase; ALT, alanine aminotransferase; Alk Phos, alkaline phosphatase

The rest of the investigations to be done and their schedules are summarized in Fig. 2.

### Research laboratory evaluations

Pharmacology evaluations will be performed as follows:

A. Plasma samples for PK analysis

Plasma samples will be collected from the first 20 subjects treated ATP in part A and for the 10 subjects in part B:

- Days 1 and 5 (1st day and last day of dosing): PK plasma samples will be collected prior to study drug administration:
  - Pre-dose: 0 h
  - Post-dose 1: 2 h (± 12 min), 3 h (± 18 min) and 4 h (± 24 min)
  - Pre-dose 2: immediately before the second dose (or skip if within 30 min of the previous sample ~ 4 h)
  - Pre-dose 3: 12 (up to 16) h post 1st dose
- Day 2 and 3 (2nd and 3rd days of dosing): PK plasma samples will be collected prior to study drug administration
- Day 6**:** PK plasma samples will be collected at approximately the time of discharge and the time noted

After discharge, the plasma PK samples will be collected on both follow up visits.

The following samples will be collected for the final diarrheic (part A) subjects, after the first 20 diarrheic subjects are enrolled and treated according to protocol.

- Day 1: 0 h (pre-dose), 4 h (± 24 min) post 1st dose
- Day 5: pre-dose 3, immediately before the third dose, and 4 h (± 24 min) post dose 3
- Day 6: just prior to discharge (note time post final dose)

B. Stool samples for PK analysis

Stool samples for PK endpoints will be collected from the first 20 part-A subjects and all part-B subjects. A stool sample for baseline CFZ levels will be collected before dosing on day 1 (first inpatient day). Total stool collection roughly every 8 hours (~ q8 h measured, scored, mixed, and sampled) will be undertaken during the days of inpatient stays throughout the study. These stools will be pooled. Stool will be collected on days 2 and 5 for measurement of CFZ levels. On day 6 the morning stool will be collected for CFZ determination prior to discharge. Subjects will be asked to return to the outpatient clinic for one outpatient follow up visit during the interval days 19–24, and one outpatient follow up visit during the interval days 41–55. The subject will be asked to bring morning stool samples with them in containers provided for qPCR Cryptosporidium excretion and CFZ levels.

## Statistical considerations

### Analysis plan

All continuous variables will be summarized using the following descriptive statistics: number (analysis population size) (*N*), number (non-missing sample size) (*n*), mean, standard deviation (SD), median, interquartile range (IQR), maximum and minimum. Note that the geometric mean will also be reported in descriptive summary tables for log-transformed variables. The proportion of observed levels will be reported for all categorical measures. When appropriate, corresponding exact 95% confidence intervals (CIs) for proportions will be included.

In general, all data will be listed, sorted by study design (part A and part B) and by subjects. When appropriate, data will also be presented by study day within study design and subject. All summary tables will be structured with a column for each treatment in the order (CFZ, placebo) for part A and a column for part B. If applicable, a column for the part A subjects that were matched to part B subjects will also be provided.

The primary analysis will be based on the ATP population. Safety analyses will be based on the safety population and all PK analyses will be based on the PK population. Analysis populations are defined below.

### Analysis populations

This study will have three analysis populations: the ITT population, the population that was treated ATP, and the PK population. The ITT population will consist of all randomized subjects. The ATP population will consist of all subjects in the ITT population who meet the following criteria: received at least one dose per day for 5 days, completed daily assessments of fecal shedding, and had no major protocol deviations. The PK population will consist of all ITT subjects who had at least one measurable PK concentration.

### Safety analysis (part A)

Cumulatively, the CFZ safety database represents more than 2 million patient-years of exposure. Toxicities seen with CFZ are most commonly observed after weeks to months of use and include skin color change (reddish/brownish) and GI symptoms (abdominal pain, constipation, with accumulation of CFZ in the GI tract). In a study recently conducted of CFZ treatment in tuberculosis (drug regimen 300 mg per day for 3 days followed by 100 mg/day for the remainder of 2 weeks), 3/60 patients on CFZ-containing regimens developed some skin discoloration while 0/45 patients on non-CFZ regimens experienced skin discoloration (D. Herman, personal communication). Rarely, hepatobiliary disorders, such as hepatitis and associated liver function test abnormalities have been reported. Some short-term GI intolerance has also been reported, but can usually be managed symptomatically without suspending administration of the drug. No hematological or biochemical blood abnormalities have been associated with CFZ use.

All safety analyses will be based on the ITT population and will be presented by treatment group. The baseline value for all subjects will be the assessment made on day 1 in the Clinical Inpatient Unit. ECG results, vital signs, laboratory parameters, and physical examination abnormalities will be presented in list format.

### Efficacy analysis (part A)

The primary efficacy endpoint in the present trial is the change from baseline in the (log) number of Cryptosporidium shed (in terms of number of oocysts shed per gram) in the first stool collected each day over a 5-day inpatient period, as measured by qPCR in treatment versus placebo. The primary analysis will be performed based on the ATP population. A mixed ANCOVA model for repeated measures will be used to model and analyze the difference between treatment groups, in the change from baseline in the log-transformed number of oocysts shed per gram in the first individual stool collected via qPCR each day over the 5-day inpatient period, starting with day 2. The model will include terms for gender, age, HIV stage of infection, the baseline log number of Cryptosporidium shed, day, and treatment group. Exploratory analyses will comprise linear regression models to consider the effects of various factors on reductions in fecal shedding, including age, sex, body weight, drug exposure, baseline oocyst shedding, parasite genotype, time on ARV treatment, CD4 count, viral load, and individual 50% minimum inhibitory concentration (MIC_50_).

### Interim analysis

A detailed description of the interim analysis plan will be included in the statistical analysis plan (SAP) that will be completed before the interim analysis. After approximately 20 subjects have completed the study ATP, an interim analysis of the primary efficacy endpoint and for futility will be performed. Alpha for the interim and final analyses will be apportioned using a power family alpha spending function with a phi value of 0.58. This alpha-spending function apportions approximately alpha = 0.03 one-sided for both the interim and final analysis, assuming an information fraction of 40%. At the time of the interim analysis the confidence interval (CI) for the difference in mean oocyst reductions over the treatment period (log2 CFZ – log2 placebo) will be determined using ANCOVA. If the upper limit of the CI is less than − 1, then the trial will be stopped early for success. If the lower limit exceeds − 1, then the trial will be stopped early for futility.

### Final analysis

Due to the interim analysis, the primary and secondary efficacy analyses will be conducted using an alpha level of 0.03 for determination of significance in a one-sided comparison. Although the test of significance will be conducted at alpha level of 0.03, the overall type I error will be 0.05 one-sided which will be used for the inference of primary and secondary efficacy outcomes.

### Secondary analyses

A Cox proportional hazards regression model will be used to evaluate whether the time to first negative fecal ELISA signal is decreased in subjects randomized to CFZ. ANCOVA will be used to assess the reduction in the number of diarrheal episodes and the (log) number of Cryptosporidium shed in stools in the first collected stool of the day over a 5-day period (days 2–6).

### Pharmacokinetic analysis (part B)

All PK analyses will be based on the PK population. Non-compartmental PK analysis will be undertaken to derive relevant PK parameters (e.g., AUC 0–24, AUC 0–8, Cmax, Cmin for day 1 (1st dose day) and day 5 (last dose day). Terminal half-life will be determined after the last dose. PK parameters will be summarized using descriptive statistics. Given the PK characteristics of CFZ, exposures are expected to accumulate over the 5-day dosing period. The accumulation ratio will be defined as AUC 16–24 on day 5 divided by the AUC 0–8 on day 1. PK parameters in symptomatic subjects will be compared to those in matched asymptomatic subjects. The total daily amount of CFZ eliminated in the feces will be calculated on the second and last dose days. Stool PK parameters from Cryptosporidium-positive subjects with diarrhea and Cryptosporidium-negative subjects without diarrhea will be compared.

## Safety monitoring

The safety of the study will be monitored by the site Principal Investigator (PI), the study Medical Monitors, an independent Local Safety Monitor (LSM), and the Data Safety Monitoring Board (DSMB). The LSM is an experienced clinician independent of the investigator team, but on the staff at QECH. The LSM will be notified within 24 h of the Site PI being aware of a SAE occurrence. A DSMB will be appointed to provide data and safety oversight. The DSMB, consisting of a minimum of three qualified members, will review all SAEs and adverse events of special interest (AESI) as cumulative reports at their scheduled meetings, or individually on an ad hoc basis, requested by the Sponsor Medical Monitor, the PI, or the LSM. The DSMB and the LSM have the power to recommend holding or stopping of the study to the Sponsor, if deemed necessary. Interim analyses on unblinded data will be performed by the DSMB after 20 subjects in part A have completed treatment ATP. The DSMB will recommend to the Sponsor whether to continue or terminate the study based on the interim analyses. The Pharmacy, Medicines and Poisons Board (PMPB) and the National Health Sciences Research Committee will be notified within 24 h of becoming aware of a SAE. All safety events observed under this protocol will be reported through the Advantage eClinical^SM^ data system. Subjects who experience AEs that are ongoing at the time of the last scheduled follow up visit will be followed to resolution or stabilization. Subjects who are pregnant during the study will be followed to completion of pregnancy.

## Discussion

Human cryptosporidiosis is typically a self-limited infection, except in malnourished children under 2 years of age and in seriously immunosuppressed patients, especially patients with HIV infection. Effective treatment of HIV infection reduces the risk of severe cryptosporidiosis, but patients with severe disease may still experience severe manifestations from Cryptosporidium infection that include wasting and death if immunosuppression cannot be alleviated with ARVs.

CFZ is an anti-leprosy agent on the WHO Essential Medicines list. It has been a Food and Drug Administration (FDA)-approved drug, although its distribution in the USA is currently limited to specialized leprosy treatment centers. CFZ is also used for treatment of multidrug resistant tuberculosis. CFZ exerts a slow bactericidal effect on *Mycobacterium leprae* by inhibiting mycobacterial growth, possibly by binding preferentially to mycobacterial DNA. It also exerts anti-inflammatory properties in controlling erythema nodosum leprosum (ENL) reactions through inhibition of plasma membrane K+ transporters, and increased production of prostaglandin E2 and the interleukin-1 (IL-1) receptor antagonist by immune, inflammatory and other cell types [8, 9]. Other mechanisms of CFZ antibacterial effect that have been proposed include membrane-directed activity on the bacterial respiratory chain and ion transporters, generation of superoxide-hydrogen peroxide, and the antimicrobial lysophospholipids, which promote membrane dysfunction, resulting in interference with K+ uptake [10–12]. CFZ also exerts its anti-mycobacterial action by its effect on the tissue macrophages, which are the main targets of infection as well as immune response in tuberculosis [13]. It is taken up by macrophages throughout the body; in autopsies performed on leprosy patients, CFZ crystals have been found predominantly in the mesenteric lymph nodes, adrenals, subcutaneous fat, liver, bile, gall bladder, spleen, small intestine, muscles, bones, and skin.

This study investigates the safety, tolerability and efficacy of orally administered CFZ in subjects with HIV infection, chronic diarrhea, and Cryptosporidium infection, and the PK of CFZ in HIV-infected adults with and without Cryptosporidium infection and diarrhea. CFZ has been shown to inhibit CYP3A4 in vitro, and therefore has the potential to lead to drug-drug interactions especially with certain antiretrovirals (ARVs) used in HIV therapy, though not with the first-line current ARV treatment used in Malawi in adults (currently tenofovir/lamivudine/efavirenz).

The strength of part A of this study lies in it being a randomized, double-blind, placebo-controlled trial. If shown to be effective and safe, findings will also lay a foundation for a future study on the use of CFZ in children 6–18 months of age. The trial is sponsored by the University of Washington and has been approved by the Liverpool School of Tropical Medicine and National Health Sciences Research Committee of Malawi (NHSRC). It will be conducted by the Malawi-Liverpool-Wellcome Trust Clinical Research Programme (MLW), in collaboration with the University of Washington and the Emmes Corporation.

There are some anticipated challenges in this study. The study has strict exclusion criteria, which may make recruitment of the subjects a challenge. We are planning to recruit patients in the outpatient department of QECH, the Gateway Clinic and in the wards. We hope this will give sufficient numbers to enroll into the study. It is anticipated that a few subjects may not remain in the clinical inpatient unit for the planned duration of the inpatient phase of the study. In these cases, an effort will be made to continue IP administration and study assessments on an outpatient basis. Subjects will be encouraged to return to the outpatient clinic for continued participation ATP. If subjects refuse to come to the outpatient clinic, an effort will be made to visit them in their homes for follow-up with regard to safety. They will be encouraged to return on day 6 for final ECG. Another challenge is that, in our experience and that of others, multiple pathogens in addition to Cryptosporidium are found in patients with HIV, Cryptosporidium infection, and diarrhea. A potential anti-Cryptosporidium drug, such as CFZ, may have no effect on the other pathogens, and thus may not alleviate symptoms. Thus we are measuring the reduction in cryptosporidium excretion as the primary outcome, and not cessation of clinical symptoms, which is a secondary outcome. Another challenge may be variability in excretion of Cryptosporidium from day to day. Since subjects will be on ARVs, there is a risk that they will self-cure. To our knowledge, no previous study has tried to quantitate Cryptosporidium excretion in the study population. Thus, it remains to be seen whether a therapeutic effect on Cryptosporidium excretion will be measureable.

In summary, this study provides an opportunity to explore a possible treatment option for HIV-infected patients with cryptosporidial diarrhea, who, as of now in Malawi and most of sub-Saharan Africa, do not have a definitive treatment apart from supportive care.

## Trial status

This manuscript presents version 4.0 of the CRYPTOFAZ protocol. The trial opened for accrual in December 2017 and is expected to be completed by October 2018.

## Additional file

Additional file 1: Standard protocol items: recommendation for interventional trials (Spirit) checklist. (DOC 190 kb)
