# Supplementary material for: Evaluating the safety, tolerability, pharmacokinetics and efficacy of clofazimine in cryptosporidiosis (CRYPTOFAZ): study protocol for a randomized controlled trial
Source: Trials. 2018 Aug 23;19:456. doi: 10.1186/s13063-018-2846-6 (PMC6108095; doi:10.1186/s13063-018-2846-6)
Supplement: Supplementary file 1 — Standard protocol items: recommendation for interventional trials (Spirit) checklist. (DOC 190 kb) [file 13063_2018_2846_MOESM1_ESM.doc]

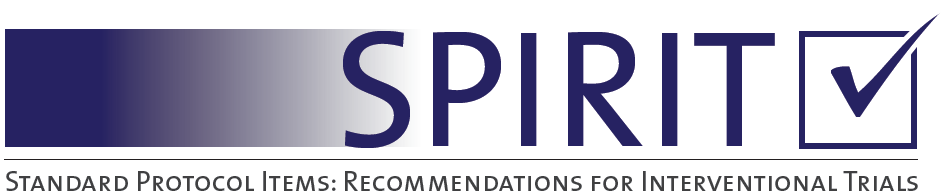


SPIRIT 2013 Checklist: Recommended items to address in a clinical trial protocol and related documents*

| Section/item | ItemNo | Description |
| --- | --- | --- |
| **Administrative information** | | |
| Title | 1 | **A Phase 2A, Randomized, Double-Blind, Placebo-Controlled Evaluation of the Safety, Tolerability, Pharmacokinetics and Efficacy of Clofazimine (CFZ) in Cryptosporidiosis (CRYPTOFAZ)** |
| Trial registration | 2a | **Trial registry name:** Clinical Trials.gov. **Number:** NCT03341767. **Date registered:** 14 Nov 2017 |
|  | 2b | **World Health Organisation Trial Registration Dataset**: See attached Table (Additional file 2) |
| Protocol version | 3 | **Protocol:** version 1.0; modified to version 2.0  **Revision chronology**  Version 2.0, 28 July 2017  Version 3.0, 7 December 2017  Version 4.0, 6 April 2018  Primary reason for amendment: modifications in recruitment, clarifications in the protocol and change in the definition of the primary endpoints (v.2.0), minor changes to the consent forms and patient information sheets (v.3.0), changes to enrollment criteria (v.4.0). |
| Funding | 4 | Funder: Bill and Melinda Gates Foundation. |
| Roles and responsibilities | 5a | **Names, affiliations, and roles of protocol contributors**  Patrick Nachipo,^1^ Dave Hermann,^2^ Gerald Quinnan,^3^ Melita Gordon,^1,4,5^ Wesley C. Van Voorhis,^6^ Pui-Ying Iroh Tam,^1,5,7*^  ^1^University of Malawi College of Medicine, Blantyre, Malawi  ^2^Bill and Melinda Gates Foundation, Seattle, WA, USA  ^3^Emmes Corporation, Rockville, MD, USA  ^4^University of Liverpool, Liverpool, United Kingdom  ^5^Malawi-Liverpool Wellcome Trust Clinical Research Programme, Blantyre, Malawi  ^6^University of Washington, Seattle, WA, USA  ^7^Liverpool School of Tropical Medicine, Liverpool, United Kingdom  **Corresponding author***  Pui-Ying Iroh Tam  Malawi Liverpool Wellcome Trust Clinical Research Programme  P.O Box 30096  Chichiri  Blantyre 3  irohtam@mlw.mw  +265 (0) 1876444  **Protocol contributors**  Grant holder: WVV  Conceived of the study: WVV, DH  Protocol development: WVV, DH, GQ, MG, PI  Implemented the trial: WVV, GQ, PN, PI  All authors contributed to refinement of the study protocol and approved the final manuscript. |
|  | 5b | The trial sponsor is University of Washington Foundation. Contact person: Claire Colson, ccolson@uw.edu. |
|  | 5c | The funder and the sponsor played no role in the study design; collection, management, analysis, and interpretation of data; writing of the report; and the decision to submit the report for publication. |
|  | 5d | **Trial Coordinating Committee**   - Members: Dr. Wes Van Voorhis (University of Washington), Elaine Douglas (University of Washington), Dr. Pui-Ying Iroh Tam (Malawi-Liverpool Wellcome Trust Clinical Research Programme), Markus Gmeiner (Malawi-Liverpool Wellcome Trust Clinical Research Programme), Neema Toto (Malawi-Liverpool Wellcome Trust Clinical Research Programme), Dr. Gerald Quinnan (Emmes Corporation), Leigh Sawyer (Emmes Corporation), Pankaj Dua (Emmes Corporation). - Agreement of final protocol. - Recruitment of study participants. - Reviewing study progress and agreeing changes to the protocol if necessary. - Reviewing adverse events.   **Data Safety and Monitoring Board**   - Independent members from the trial investigators. Steven Reynolds (Chair), NIAID/Uganda; Jane Mallewa, University of Malawi College of Medicine; David Boulware, University of Minnesota; David Lalloo, Liverpool School of Tropical Medicine. - Reviewing interim analysis results including safety data. - Unblinding.   **Medical Monitor**   - Frederick Buckner (University of Washington); Gerald Quinnan (Emmes Corporation). - Review safety issues that arise during the study.   **Independent statistician**   - Dr Maia Lesosky (University of Cape Town). - Responsible for randomisation and code breaking. |
| Introduction |  |  |
| Background and rationale | 6a | Research question: Is clofazimine safe, tolerable, and effective in treating *Cryptosporidium* diarrhea?  *Cryptosporidium* infection and diarrhea (cryptosporidiosis) is a life-threatening infection in persons with HIV and also in children of 6-18 months of age in the developing world. To date, only nitazoxanide is licensed for treatment of cryptosporidiosis, and only in persons after the first year of life and with healthy immune systems. Clofazimine (CFZ: Lamprene®), an old drug which has been used for leprosy for more than 50 years, recently has been described as effective against *Cryptosporidium* *in vitro* and in mouse infections. *In vivo,* the efficacy and pharmacokinetics of CFZ in HIV-infected patients with cryptosporidial diarrhea are not known. |
|  | 6b | **Comparator:** Placebo |
| Objectives | 7 | Part A Primary objective   1. To evaluate whether there is a reduction in *Cryptosporidium* fecal shedding following CFZ administration relative to placebo in HIV-infected adults, with diarrhea and *Cryptosporidium* infection. 2. To investigate the safety and tolerability of CFZ when orally administered daily for 5 consecutive days to HIV-infected adults with diarrhea and *Cryptosporidium* infection.   Secondary objectives   1. To evaluate whether the time to negative fecal enzyme linked immunosorbent assay (ELISA) signal is decreased in subjects randomized to CFZ. 2. To characterize the reduction in the number of diarrheal episodes, stool volume and consistency following administration of CFZ relative to placebo. 3. To characterize the fecal shedding of *Cryptosporidium* and symptomatology of cryptosporidiosis in subjects receiving placebo for future therapeutic trials.   Part B Primary objective  To investigate the pharmacokinetics of CFZ in HIV-infected adults with *Cryptosporidium* infection and diarrhea and compare to the pharmacokinetics from HIV-infected subjects without *Cryptosporidium* infection or diarrhea. |
| Trial design | 8 | This is a Phase IIa randomized, double-blind placebo-controlled study of the safety, tolerability and efficacy of orally administered CFZ in subjects with HIV infection, chronic diarrhea, and *Cryptosporidium* infection. |
| Methods: Participants, interventions, and outcomes | | |
| Study setting | 9 | The study will recruit in A&E and inpatient wards at Queen Elizabeth Central Hospital, and outpatient clinics in Blantyre district, Malawi. |
| Eligibility criteria | 10 | **Inclusion criteria**   1. Passed prescreening for PART A (Male or Female, Aged 18 to 65 years old, HIV positive, *Cryptosporidium* positive by qPCR, and on stable anti-retroviral (ARV) treatment for at least 2 weeks). 2. Weight >78 lbs/35.4 kg 3. Presents with chronic diarrhea defined as three or more loose stools per day that has persisted for 3 days or longer. (If subjects have blood in their stools, they will be treated with Ciprofloxacin which is the standard of care, but will not be excluded.) 4. If female, either not of reproductive potential (post-menopause, or status post-surgical sterilization) or using highly effective contraception (<1% failure, e.g. intrauterine contraceptive device in place or using injectable contraception) or willing to begin highly effective contraception (probably injectable contraception) and continue for the presumed exposure period of CFZ (54 days after initiation of IP administration). 5. Willing and able to provide signed written IC or witnessed oral consent in the case of illiteracy, prior to undertaking any study-related procedures.   **Exclusion criteria**   1. Any condition for which participation in the trial, as judged by the Site Investigator, could compromise the well-being of the subject or prevent, limit or confound protocol specified assessments. 2. Fever >38.0˚C on presentation. 3. Evidence of active tuberculosis based on acid fast bacilli staining or GeneXpert testing of sputum OR sputum production, fever, and chest Xray consistent with tuberculosis. 4. Is critically ill or in the judgment of the investigator has a prognosis that could lead to imminent mortality within 60 days or compromise participation in the study or endanger the patient by entering the study. 5. Has a history of allergy or hypersensitivity to CFZ. 6. Significant cardiac arrhythmia requiring medication. 7. Exclusions based on ECG: Subjects with the following at Day -1 (where applicable to ECGs, the mean of the triplicate automated ECG result to be used), as exclusions: 8. Marked prolongation of QT/QTc interval, e.g., confirmed demonstration of QTcF or QTcB interval >450 milliseconds (ms). 9. Pathological Q waves (defined as >40 ms or depth >0.4 millivolt [mV]). 10. ECG evidence of ventricular pre-excitation. 11. ECG evidence of complete or incomplete left bundle branch block or right bundle branch block. 12. ECG evidence of second or third degree heart block. 13. Intraventricular conduction delay with QRS duration >120 ms. 14. Bradycardia as defined by sinus rate <50 bpm. 15. Use of concomitant medications that markedly prolong the QT/QTc interval or are predicted to have drug-drug interactions with CFZ that may lead to toxicity from the partner drug. 16. History of additional risk factors for Torsade de Pointes, e.g., heart failure, bradycardia (HR<50 bpm), untreated hypothyroidism. 17. Family history of long QT syndrome. 18. Pregnant and lactating women (screening pregnancy test for females). 19. Use of systemic corticosteroids or anti-cryptosporidial treatments within the 28 days preceding the screening visit. 20. Subjects with clinically significant laboratory value abnormalities at eligibility screening (note: exclusionary results should be confirmed by the time of the beginning of administration of IP, as described in the study Manual of Procedures):  - Haemoglobin <5 g/deciliter (dL) - Serum potassium <3.2 milliequivalents per liter (mEq/L)  1. Aspartate aminotransferase (AST) or Alanine aminotransferase (ALT) ≥3.0 x upper limit of normal (ULN) |
| Interventions | 11a | 17. Family history of long QT syndrome.   1. Pregnant and lactating women (screening pregnancy test for females). 2. Use of systemic corticosteroids or anti-cryptosporidial treatments within the 28 days preceding the screening visit. 3. Subjects with clinically significant laboratory value abnormalities at eligibility screening (note: exclusionary results should be confirmed by the time of the beginning of administration of IP, as described in the study Manual of Procedures): 4. Haemoglobin <5 g/deciliter (dL) 5. Serum potassium <3.0 milliequivalents per liter (mEq/L) 6. Aspartate aminotransferase (AST) or Alanine aminotransferase (ALT) ≥3.0 x upper limit of normal (ULN)   Intervention arm 1: Clofazimine, 100 mg three times daily (TID) for subjects ≥50 kg; Clofazimine 50 mg TID for subjects <50 kg, for 5 days.  Intervention arm 2: Placebo, TID orally, for 5 days. |
|  | 11b | There will be no change in dosing during the trial. Criteria for discontinuing the study drug will be: Two or more subjects with a Suspected Unexpected Serious Adverse Reaction (SUSAR) occurring within six days following the initiation of treatment.  Three or more subjects experience the same Grade 3 laboratory abnormality, assessed as clinically significant worsening related to the IP, within 6 days following initiation of treatment. |
|  | 11c | Participants will receive the study drug through directly observed therapy (DOTs) by a designated caregiver. Administration of the study drug will be reflected on patient case report forms. The study team will count remaining pills at each study visit. |
|  | 11d | During the course of the study concomitant use of known drugs that prolong QTc interval will be avoided, including: Amiodarone, Amprenavir, Atazanavir, Bedaquiline, Bepridil, Chloroquine, Chlorpromazine, Cisapride, Clarithromycin, Cyclobenzaprine, Darunavir, Delamanid, Disopyramide Dofetilide, Domperidone, Droperidol, Erythromycin, Fosamprenavir, Halofantrine, Haloperidol, Ibutilide, Indinavir, Levomethadyl, Lopinavir, Mesoridazine, Methadone, Nelfinavir, Pentamidine, Pimozide, Procainamide, Quinidine, Ritonavir, Simiprinivir, Sotalol, Sparfloxacin, Thioridazine, or Tiprinivir. |
| Outcomes | 12 | Primary Endpoints:   1. Safety assessments collected throughout follow up period: AEs, serious adverse events (SAEs), vital signs, 12-lead electrocardiogram (ECG), physical examinations (PE), and clinical laboratory evaluations. 2. The reduction in the (log) number of *Cryptosporidium* shed in stools in the first collected stool of the day over a 5-day period and compared to placebo recipients, as measured by qPCR in stool samples and analyzed by a mixed effect ANCOVA analysis in subjects treated ATP (PART A only). 3. Pharmacokinetics of CFZ in plasma: area under the curve (AUC), peak plasma concentration (Cmax), and time to reach Cmax (Tmax) for second and last dose days; half life (T ½) determined after last dose.   Secondary Endpoints:   1. The reduction in the (log) number of *Cryptosporidium* shed in stools in the first collected stool of the day over a 5-day period and compared to placebo recipients in the ITT subject population. 2. The reduction in total daily *Cryptosporidium* shedding over a 6-day period in subjects treated ATP (PART A only). 3. The reduction in total daily *Cryptosporidium* shedding over a 6-day period when in comparison to placebo controls in the ITT population. 4. The reduction in severity of diarrhea over a 6-day period in comparison to placebo controls in subjects treated ATP. |
| Participant timeline | 13 | | **Study Day/Period^1^** | **Activities** | | --- | --- | | Prescreen | Prescreening informed consent (IC) Confirmation of HIV status (review of records) Rapid Diagnostic Test (RDT), quantitative polymerase chain reaction (qPCR) for *Cryptosporidium* | | Enrollment | Study participation IC  Screening tests performed Eligibility evaluation | | -1 | Eligibility confirmation Admission to the Clinical Inpatient Unit Approximately every 8 hour (hr) (~q8 hr) stool measurement and sampling, baseline run-in | | 1 | Randomization First dose of investigational product (IP)  ~q8 hr stool measurement and sampling, and assessments | | 2-5 | IP administration, ~q8 hr stool measurement and sampling, and assessments | | 6 | Post administration of IP assessments Stool collection in the morning prior to discharge  Discharge from Clinical Inpatient Unit | | 19-24 | One Outpatient Follow Up Visit during interval | | 41-55 | One Outpatient Follow Up Visit during interval | | ^1^Entire process from prescreen consent to randomization on Day 1 will take from 1 to 5 days | | |
| Sample size | 14 | PART A:  This is a Phase 2a study, and is largely exploratory. The sample size for PART A is planned based on the goal that demonstration of a significant inhibition of *Cryptosporidium* shedding should be possible in a relatively small number of subjects, if the drug is to be worth further development efforts. Based on results in a calf model with a controlled challenge, the sample size required might be very small (e.g., 10 per group), if the variability of shedding in the study population planned for enrollment in this study is small. Since the variability in shedding in the planned study population cannot be estimated, a study size of 25 per group (increased to 28 per group to cover drop-outs) is planned in case variability is more substantial than expected. An interim analysis is planned after 20 subjects have been randomized and successfully completed the IP administration phase of the study. An analysis of the primary endpoint is planned at that point for significance. If significance is obtained, the study will be terminated. If significance is not obtained, an analysis for futility of continuation will be performed. If the study appears to be adequately powered to achieve significance of the primary endpoint based on that analysis, it will be continued until full enrollment and follow‑up.  PART B:  This part of the study is empirical. Pharmacology of CFZ will be compared in the individuals receiving CFZ in PART A with those individuals receiving CFZ in PART B. The objective is to develop a comparative description of the absorption and excretion of the drug in the HIV positive populations with and without *Cryptosporidium* associated diarrhea. The primary analysis will include the first 10 subjects randomized to CFZ in PART A and the 10 subjects receiving CFZ in PART B. |
| Recruitment | 15 | Strategies for achieving adequate participant enrolment to reach target sample size include one-one information about the study and presence of the study team in as many outpatient clinics as possible. |
| **Methods: Assignment of interventions (for controlled trials)** | | |
| Allocation: |  |  |
| Sequence generation | 16a | The allocation sequence was obtained using computer-generated random numbers, Randomization of subjects will be done online using the enrollment module of Advantage eClinical. The randomization code will be prepared by statisticians at the Emmes Coporation and included in the enrollment module for this trial. |
| Allocation concealment mechanism | 16b | Advantage eClinical will assign each subject to a group after the demographic and eligibility data have been entered into the system. The coded treatment assignments will be included in the enrollment module for the trial for PART A only. About 56 subjects enrolled (who will be continuing to treatment based on results of screening tests) in the trial will be assigned to a coded treatment assignment on Study Day 1. Subject randomization will occur at the time of initiation of treatment. |
| Implementation | 16c | Enrollment of subjects for PART A and B will be performed online using the enrollment module of Advantage eClinical. Once consented and upon entry of demographic data and confirmation of eligibility for this trial, the subject will be enrolled. |
| Blinding (masking) | 17a | This is a double blinded trial. The unblinded pharmacist will be provided with the treatment assignment codes for preparation of the CFZ or placebo to be given to each subject. The research pharmacist will not reveal the randomization code to any other study staff member or subject. The research pharmacist will dispense the appropriate study product (drug or placebo) in a blinded manner. The treatment assignment of those subjects randomized in PART A will remain blinded to the clinical team until database lock, as defined in the SAP. |
|  | 17b | Unblinding will be allowed in case of emergency during the course of the study. A designated individual at the site, i.e. the research pharmacist, will be provided with a code list for emergency unblinding purposes, which will be kept in a secure place. |
| **Methods: Data collection, management, and analysis** | | |
| Data collection methods | 18a | Data collection is the responsibility of the clinical trial staff at the study site under the supervision of the Site Investigator. Data for each subject will be recorded in the hard copy CRFs and verified by the Site Investigator. Consistency checks and checks for missing data will be performed at data entry and after the database has been merged. |
|  | 18b | In order to retain participants and to complete follow-up of participants we plan to trace participants in the first 24 hours of missing an appointment, if contact is not possible by phone the study team will visit the participant’s home. |
| Data management | 19 | Data Management activities will be provided by Emmes with cooperation from MLW data management. Data will be transferred on a regular basis to the main data management hub at Emmes through a secured link. Data will be backed up daily at each site.  Source documents and paper based report forms will be stored in a locked filling cabinet separate from any participant’s identifier. |
| Statistical methods | 20a | PART A of the study will be assessed in a final analysis and will also have the primary efficacy endpoint (i.e., the reduction in the (log) number of *Cryptosporidium* shed in the first collected stool of each day through Day 6 in CFZ *versus* placebo recipients in subjects treated per protocol) and safety and futility assessed in an interim analysis after 20 subjects have data reported through Day 6. PART B of the study will be assessed in one final analysis.  This study will have three analysis populations: the intention-to-treat (ITT) population, the according to protocol (ATP) population, and the pharmacokinetic (PK) population. The ITT population will consist of all randomized subjects. The ATP population will consist of all subjects in the ITT population who meet the following criteria: received at least one dose per day for five days, completed daily assessments of fecal shedding, and had no major protocol deviations. The PK population will consist of all ITT subjects who had at least one measurable PK concentration. Safety Analysis (PART A)  - All safety analyses will use the ITT population and will be presented by treatment group. The baseline value for all subjects will be the assessment taken on Day 1 in the Clinical Inpatient Unit. - The number, percentage, and exact two-sided 95% confidence interval (CI) for subjects reporting each solicited AE will be summarized by severity and day. Additionally, the maximum severity of each event experienced by each subject will be summarized. - The number, percentage, and exact two-sided 95% CI for subjects reporting at least one unsolicited AE throughout the course of the study will be summarized by MedDRA® System Organ Class (SOC) and Preferred Term (PT). In addition, AEs will be summarized by relatedness and severity. In addition, unsolicited AEs will be summarized by relatedness and severity. - SAEs, SUSARs, and other clinically significant events will be presented in listing format, including MedDRA® SOC and PT. - ECG results, including overall interpretation, interval measurements, and any findings, will be summarized by time point and a complete listing of results will be presented. - Vital signs and laboratory parameters will be summarized categorically by severity and continuously by change from baseline for each time point assessed. In addition, abnormal results will be listed. - Any abnormalities noted upon physical examination will be presented in listing format. - The number and percentage of subjects who discontinue treatment for any reason in each group will be compared *via* Fisher’s Exact Test. |
|  | 20b | Efficacy Analysis (PART A) The primary efficacy endpoint in the present trial is the reduction in the (log) number of *Cryptosporidium* shed in the first collected stool collected after dose 1 each day, as measured by qPCR in treated *versus* placebo recipients treated ATP. Total stool volume will be determined as the sum of the weights of each stool assuming each g is equal to one mL. The total number of *Cryptosporidium* shed in stools over a 24 hr period will be the sum of the number of *Cryptosporidium* measured across all stools on the given study day. The primary analysis will be performed using the ATP population and will compare subjects receiving CFZ to subjects receiving placebo in terms of the reduction of *Cryptosporidium* shedding over the 6-day period using mixed effects ANCOVA analysis with multiple imputation for missing data. Any day of days 1-6 in which a fecal sample is not collected for testing will be considered a missing data point. The covariates in the model for testing the primary endpoint will be gender, age, HIV grade of infection, (log) number of *Cryptosporidium* shed in the first collected stool collected on day -1 and 1 (baseline) and treatment group. All study participants are expected to be Bantu, so race and ethnicity will not be included as covariates. The first fecal samples with no detectable *Cryptosporidium* will be imputed to ½ the lower limit of detection. Consecutive samples with no detectable *Cryptosporidium* will be imputed to log transformed values of zero**.** An analysis of the primary efficacy endpoint using the ITT analysis population will be performed as a secondary analysis. Exploratory analyses will use linear regression models to consider the effects of various factors on reductions in fecal shedding including age, sex, body weight, drug exposure, baseline oocyst shedding, parasite genotype, time on ARV treatment, CD4 count, viral load, and individual MIC_50_.  Any variables that show imbalances will be adjusted for when analysing the trial outcomes at the end of the second stage using logistic regression with random effects. ***Interim Analysis***  Detailed description of the interim analysis plan will be included in the SAP that will be completed before the interim analysis. After approximately 20 subjects have completed the study ATP, an interim analysis of the primary efficacy endpoint and for futility will be performed. Alpha for the interim and final analyses will be apportioned using a power family alpha spending function (1) with a phi value of 0.58. This alpha-spending function apportions approximately alpha = 0.03 one sided for both the interim and final analysis, assuming an information fraction of 40%. At the time of the interim analysis the CI for the difference in mean oocyst reductions over the treatment period (log_2_ CFZ – log_2_ placebo) will be determined using an ANCOVA.  If the upper limit of the CI is less than -1, then the trial will be stopped early for success.  If the lower limit exceeds -1, then the trial will be stopped early for futility. |
|  | 20b  20c | ***Final Analysis***  Due to the interim analysis, the primary and secondary efficacy analyses will be conducted using an alpha level of 0.03 for determination of significance in a one-sided comparison. Although the test of significance will be conducted at alpha level of 0.03, the overall type I error will be 0.05 one-sided which will be used for the inference of primary and secondary efficacy outcomes.  ***Secondary Analyses***   - To evaluate whether the time to first negative fecal ELISA signal is decreased in subjects randomized to CFZ: A participant who does not have a negative fecal ELISA signal during the 6-day period will be right censored. A Cox proportional hazards regression model will be used to evaluate the difference in the two treatment groups. - To characterize the reduction in the number of diarrheal episodes, stool volume and consistency following administration of CFZ relative to placebo: An ANCOVA analysis will be used to assess the reduction in the number of diarrheal episodes, adjusting for the baseline (day -1) diarrheal episodes. The volume will be calculated from the weight, assuming 1 gram (g) = 1 milliliter (mL). The weight will be measured 3 times on a given day. The reduction in stool volume analysis will be conducted using ANCOVA approach adjusting for the baseline characteristic. - The reduction in the (log) number of *Cryptosporidium* shed in stools in the **first collected stool** of the day over a 5-day period (day 2 -6): Adjusting for first baseline collected stool on day -1 and 1, reduction will be analyzed using an ANCOVA model. The comparison between the CFZ and placebo recipients in the **ITT** subject population will be conducted. - The reduction in **total daily** *Cryptosporidium* shedding over a 6-day period in ATP population. - The reduction in **total daily** *Cryptosporidium* shedding over a 6-day period when in comparison to placebo controls in the **ITT** population. - The reduction in severity of diarrhea over a 6-day period in comparison to placebo controls in subjects treated ATP.   Multiple imputation will be used, if needed, in case of missing data. |
|  | | |
| **Methods: Monitoring**  Data monitoring | 21a | Data Safety and Monitoring Board (guided by Damocles Charter)   - Independent members from the trial investigators. Steven Reynolds (Chair), NIAID/Uganda; Jane Mallewa, University of Malawi College of Medicine; David Boulware, University of Minnesota; David Lalloo, Liverpool School of Tropical Medicine. - Performing interim analysis including safety data. |
|  | 21b | First interim analysis will be done after recruiting first 20 participants or earlier if there are any safety concerns. Criteria for stopping the trial will be futility or safety concerns in the intervention arm. |
| Harms | 22 | Potential risks include an allergic reaction or adverse reaction to the medication or placebo. Examples of potential side effects include nausea, vomiting, diarrhea, brownish skin discolouration and conjunctival and corneal discolouration. Each of these risks and any other unexpected outcomes will be monitored during hospital stay and at each study visit. Serious adverse events will be reported the Emmes Corporation within 24 hr of their occurrence and/or the site’s knowledge of the event. The Malawi Pharmacy, Medicines and Poisons Board (PMPB) will also be notified by MLW Clinical Research Support Unit (CRSU). |
| Auditing | 23 | The trial will be monitored by The Emmes Corporation and MLW CRSU in Malawi. |
| Ethics and dissemination | | |
| Research ethics approval | 24 | Ethics approval was obtained locally from the National Health Science Research Committee (17/05/1821), PMPB (PMPB/CTRC/2A/CFZ-001), and the Liverpool School of Tropical Medicine (17-031). |
| Protocol amendments | 25 | Important protocol modifications (eg, changes to eligibility criteria, outcomes, analyses) will be communicated first to IRBs for approval before implementation. |
| Consent or assent | 26a | Research nurses will obtain informed consent and informed consent and assent in case of participants < 18 years. All trial participants will give written or witnessed (with thumb print for illiterate participants) consent before undergoing any trial procedures. |
|  | 26b | Additional consent will be obtained for collection and use of participant data and biological specimens in ancillary studies. |
| Confidentiality | 27 | Only authorised personnel will handle the study data with password protection of both the computer and the study database. Final data will be fully anonymised to remove any participant identifying information to uphold confidentiality. |
| Declaration of interests | 28 | All principal investigators declare no other competing interests for the overall trial and each study site. |
| Access to data | 29 | All trial investigators and DSMB members will have access to the final trial dataset. There are no contractual agreements that limit access for investigators. The final fully anonymised data from the study will be made available from the corresponding author on reasonable request. |
| Ancillary and post-trial care | 30 | There are no ancillary and post-trial care. The study is covered by no-fault insurance policy for compensation to those who suffer harm during trial participation. |
| Dissemination policy | 31a | Findings will also be presented at peer-reviewed regional and international conferences. |
|  | 31b | ICMJE authorship eligibility guidelines will be followed during publication. |
|  | 31c | The final fully anonymised data from the study will be made available from the corresponding author on reasonable request. |
| Appendices |  |  |
| Informed consent materials | 32 | Model consent form and other related documentation given to participants and authorised surrogates (See attached Information sheet and consent form) |

*It is strongly recommended that this checklist be read in conjunction with the SPIRIT 2013 Explanation & Elaboration for important clarification on the items. Amendments to the protocol should be tracked and dated. The SPIRIT checklist is copyrighted by the SPIRIT Group under the Creative Commons “[Attribution-NonCommercial-NoDerivs 3.0 Unported](http://www.creativecommons.org/licenses/by-nc-nd/3.0/)” license.
